# Current european regulatory perspectives on insulin analogues

**DOI:** 10.1186/1758-5996-3-14

**Published:** 2011-07-07

**Authors:** Harald G Enzmann, Martina Weise

**Affiliations:** 1 Bundesinstitut für Arzneimittel und Medizinprodukte, Kurt-Georg-Kiesinger-Allee 3, D-53175 Bonn, Germany

**Keywords:** Insulin analogues, cancer, marketing authorization, guideline, European Medicines Agency, health technology assessment

## Abstract

Insulin analogues are increasingly considered as an alternative to human insulin in the therapy of diabetes mellitus. Insulin analogues (IAs) are chemically different from human insulin and may have different pharmacokinetic or pharmacodynamic properties. The significance of the modifications of the insulin molecule for the safety profile of IAs must be considered. This review describes the regulatory procedure and the expectations for the scientific content of European marketing authorization applications for innovative IAs submitted to the European Medicines Agency. Particular consideration is given to a potential cancer hazard. Specific regulatory guidance on how to address a possible carcinogenic or tumor promoting effect of innovative IAs in non-clinical studies is available. After marketing authorization, the factual access of patients to the new product will be determined to great extent by health technology assessment bodies, reimbursement decisions and the price. Whereas the marketing authorization is a European decision, pricing and reimbursement are national or regional responsibilities. The assessment of benefit and risk by the European Medicines Agency is expected to influence future decisions on price and reimbursement on a national or regional level. Collaborations between regulatory agencies and health technology assessment bodies have been initiated on European and national level to facilitate the use of the European Medicines Agency's benefit risk assessment as basis on which to build the subsequent health technology assessment. The option for combined or joint scientific advice procedures with regulators and health technology assessment bodies on European level or on a national level in several European Member States may help applicants to optimize their development program and dossier preparation in regard of both European marketing authorization application and reimbursement decisions.

## Introduction

Subcutaneous insulin remains the backbone for the treatment of diabetes mellitus in Europe when oral antidiabetic medicinal products are not effective or no longer sufficient. Alternative application routes for insulin have been attempted for a long time to avoid inconvenient subcutaneous injections. An inhalative formulation of human insulin has been licensed in 2006 but failed economic success, was suspected to increase lung cancers in smokers and was finally withdrawn from the market [1]. At variance with the human insulin formulation for inhalation, several IAs have been successfully introduced on the European market. In an IA, the human insulin molecule has been altered to modify its pharmacokinetic and/or pharmacodynamic properties. The development of new IAs continues regardless of an ongoing discussion on the safety of these products [2,3]. Most particularly a possible carcinogenic effect of IAs has been considered and since 2001 special recommendations of the European Medicines Agency are available on how to assess a possible carcinogenic potential of a new IA during development [4].

### General legal and regulatory requirements for innovative medicinal products

For the demonstration of efficacy, generally two pivotal studies are expected. The studies should be compliant to the principles of good clinical practice, prospective, double-blind, randomized and statistically powered not only for efficacy but also for the detection of more frequently occurring adverse effects. Superiority to an active comparator is most welcome, but a non-inferiority design to an active comparator is sufficient. Whether to include a placebo arm in form of a three arm study design or whether possibly to apply for a European marketing authorization based on superiority to a placebo alone needs careful consideration.

Whereas efficacy is defined exclusively clinically, for the assessment of the safety profile both the clinical safety data and the non-clinical studies on toxicology and safety pharmacology are important. In indications with, like diabetes mellitus, several well established treatment options, the safety profile of a medicinal product is paramount. In this case, it may not be sufficient to demonstrate that the beneficial effects of the new product outweigh its unfavourable effects. If medicinal products with similar efficacy are available, the safety profile of the new product should not be inferior to an established treatment option. Since a head to head comparison with an established treatment is best for a comparative assessment of efficacy and safety it is generally advisable to include a state of the art active comparator in a European marketing authorization application.

Economic considerations, the question of the price of a new medicinal product or whether it will be reimbursed by health care systems have no part in the marketing authorization decision that will be based on efficacy, safety and pharmaceutical quality alone. All stakeholders, however, need to keep in mind that the ultimate goal of any development is the use of the new product by the patients. This will be greatly influenced by economic factors, e.g. reimbursement decisions. The head to head comparison with a standard treatment licensed in Europe is highly recommended both for the marketing authorization application and for the subsequent assessment for pricing and reimbursement decisions. It seems advisable that comparative data required by health technology assessment bodies are generated as early as possible, e.g. within in the trials performed to support of the European marketing authorization application.

Health technology assessment bodies and decision makers on reimbursement and pricing are organized nationally or even regionally. For example, there are 7 different regional health technology assessment bodies in Spain (Agencia de Evaluación de Tecnologías Sanitarias de Andalucía; Instituto Aragonés de Ciencias de la Salud; Agència d'Avaluació de Tecnologia i Recerca Mèdiques de Catalunya; Axencia de Avaliación de Tecnoloxías Sanitarias de Galicia; Agencia Laín Entralgo de Madrid, Unidad de Evaluación de Tecnologías Sanitarias, Comunidad de Madrid; Osasun Teknologien Ebaluazioko Zerbitzua, País Vasco; Servicio de Evaluación del Servicio Canario de Salud) in addition to the national Spanisch agency Instituto de Salud Carlos III-ISCIII [5].

Marketing authorizations for a medicinal product in Europe may be obtained through national procedures in the various member states with the subsequent option of mutual recognition procedures in other member states, through the Decentralized European Procedure in member states selected by the applicant or through the European Centralized Procedure. A successful marketing authorization application in the Centralized Procedure will result in a marketing authorization by the European Commission for all member states of the European Union and the countries of the European Economic Area Iceland and Norway. Since 2004, all innovative medicinal products with the indication diabetes mellitus and all biotechnology medicinal products are in the mandatory scope of the Centralized Procedure. IAs, therefore, need to use the Centralized Procedure.

## The Centralized Procedure for Marketing Authorization in Europe

For the Centralized Procedure, the marketing authorization application is submitted to the European Medicines Agency in London, which is in the centre of the European network of Regulatory Agencies and based on the scientific expertise of about 4000 scientists at 44 regulatory agencies all over Europe. The European Medicines Agency is responsible for the selection of the assessment teams with the best available expertise for the function of Rapporteur and Corapporteur and for the procedural management. The scientific assessment is performed by the Rapporteur's and Corapporteur's multidisciplinary teams at the national regulatory agencies in the member states of the European Union. The final recommendation whether to grant a marketing authorization is consolidated in the European Medicines Agency's scientific Committee for Medicinal Products for Human Use.

**Figure 1:**
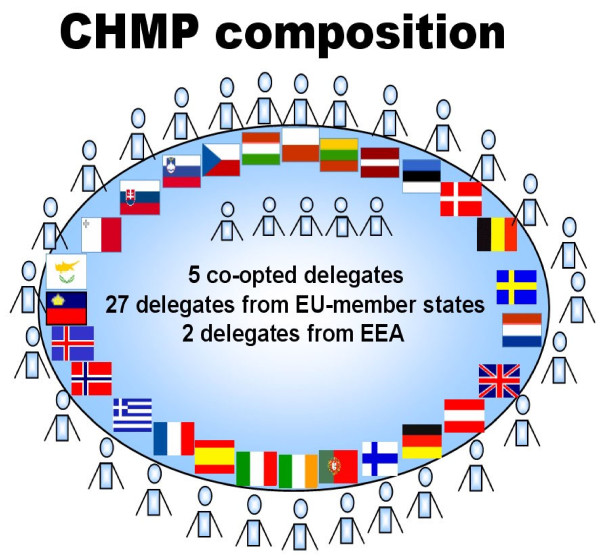
**Co-opted members**: members who provide additional expertise in a particular scientific area - i.e.: medical statistics; pharmacovigilance, pharmacoepidemiology; quality (non-biologicals), quality and safety (biological), with expertise in advanced therapies (gene, cell and tissue therapies). Delegates: CHMP members and alternates nominated by each of the EU-member states in consultation with the EMA Management Board. EEA: European Economic Area: Member States of the EU + 2 delegates from Iceland and Norway.

The 27 member states of the European Union send one delegate each to this committee. These elect five co-opted members to provide additional expertise on particularly relevant areas and each of the additional members of the European Economic Area Iceland and Norway send a delegate (Figure 1). In monthly meetings, the committee members discuss the marketing authorization application and try to reach a consensus position. If no consensus is possible a recommendation based on a majority of at least 17 votes from delegates from the European Union member states or co-opted members will be given to the European Commission.

## Demonstration of Efficacy and Safety in a European Marketing Application for innovative IAs

A European marketing authorization application for an innovative IA must include the demonstration of clinical efficacy and safety. Glycated haemoglobin (HbA1c) is an accepted surrogate parameter for treatment efficacy although clinical endpoints, e.g. cardiovascular outcome, will be very welcome. If HbA1c is used as an endpoint in a superiority design, it should be made obvious that the treatment-induced change, the difference in HbA1c between the group using the new medicinal product and the reference group is not only statistically significant but also clinically relevant. If HbA1c is used as an endpoint in a non-inferiority design, the non-inferiority margin needs to be justified to ensure that any difference in treatment effect within this margin is of no clinical relevance [6]. This is important since a glucose control even slightly inferior compared to the established treatment might result in less favourable outcome over a long time. Any potential or observed inferior efficacy should be set in relation to other possible advantages of the innovative medicinal product, e.g. with regard to safety such as lower risk of hypoglycaemia, favourable effect on body weight or improvement of patient compliance.

With several established treatment options available for diabetes mellitus including various human insulin preparations and IAs and the expectation of long term use of a new IA, safety aspects are crucial. Amongst others, data supporting the cardiovascular safety of the new antihyperglycaemic agent are expected. Regulators are very aware of the importance of this endpoint, especially since the suspension of the marketing authorisations for the rosiglitazone-containing anti-diabetes medicines [7]. These products exhibited a seemingly beneficial effect on glucose control that did not translate into an improved cardiovascular outcome but, to the contrary, data even suggested cardiovascular harm. According to current guidelines, clinical safety studies should cover a time period of at least 6 months. If a paediatric indication is applied for, a time period of at least two years should be used, looking for signals of immunogenicity or carcinogenicity. With the imminent revision of the European Medicines Agency's guideline on medicinal products in the treatment of diabetes mellitus it is expected that clinical trials of at least 18 to 24 months will be requested prior to granting a European marketing authorization. As it is obvious that an 18 to 24-month follow-up is most likely completely insufficient to detect a possible increase in tumours the matter of carcinogenicity needs to be thoroughly addressed in non-clinical studies.

## Studies on the potential cancer hazard of IAs

After the discovery of the potent carcinogenic effect of AspB10 in rats the European Medicines Agency issued a detailed guidance on what should be done to exclude or minimize the carcinogenic hazard for new IAs. In the "Points to consider document on the non-clinical assessment of the carcinogenic potential of insulin analogues" [4]*in vitro *and *in vivo *studies are described that may be appropriate to address the possible carcinogenicity of a new IA. In this document, an IA-induced increase in cell proliferation mediated by cross-reactivity with insulin-like growth factor 1 (*IGF-1*) at the IGF1 receptor (IGF1R) was considered the most likely mechanism. The statement: "... *insulins with an increased mitogenic effect in comparison to the native human insulin in current use would constitute a major public health concern*" makes clear that this hazard of IAs is taken most seriously. From today's perspective, almost ten years after adoption of the guidance document, this position may seem over-cautious. It is uncertain whether a new IA with such effects on cell proliferation could today or in the future get a positive recommendation for marketing authorization by the European Medicines Agency's Committee for Medicinal Products for Human Use. For IAs already licensed, however, effects on proliferation that have been seen in non-clinical experiments in combination with ultimately inconclusive epidemiological studies on the occurrence of cancer in humans were not considered sufficient for a suspension or withdrawal of the European marketing authorization by the European Medicines Agency. Insulin glargine has been shown to act stronger than human insulin on cell proliferation in selected breast cell lines, [8,9] in colon cancer cell lines [10], and in human lung fibroblasts [11]*in vitro*. Some epidemiological studies seemed to suggest an increased incidence of overall cancer [12], or breast cancer [13] in patients treated with an IA, while other clinical studies failed to find an increase in cancer [14] or the authors concluded that the observed increase in breast cancer reflected allocation bias rather than an effect of insulin glargine [15]. The best way to overcome these inherent limitations of epidemiological studies would be a well designed, prospective randomized controlled trial. However, the long time required to obtain meaningful resultson the occurrence of cancer in such a trial and the ethical problems of a "carcinogenicity" study in humans [16] make this approach unfeasible. Therefore, any regulatory decision will be based on a wholistic view integrating epidemiological observational data as well as preclinical studies on possible mechanisms. Overall, data on the effect of insulin glargine on the development of tumours are mostly considered as inconclusive and a change of treatment recommendations or regulatory action seem not justified to experts in the USA [17] or in Europe [2,18,19].

Whereas the interaction of IAs with the IGF1R is considered the predominant mechanism for the induction of cancer, the European Medicines Agency's "points to consider document" [4] does not exclude other possible mechanisms: *"Although enhanced Insulin-like Growth Factor 1 (IGF-1) receptor activation and/or aberrant signalling through the insulin receptor have been implicated, the mechanism(s) responsible for the mitogenic activity remain to be clarified." *The European Medicines Agency rather emphasize the need for further mechanistic studies: *"The exact mechanism(s) behind the increased carcinogenic and/or mitogenic potential of some insulin analogues remain to be elucidated"*. Consequently, the non-clinical studies on carcinogenicity of IAs are not to be restricted to IGF1R-mediated effects but "...interaction *with growth factor receptors and mechanisms other than those for IGF-1 need to be taken into account." *To minimize the hazard of a carcinogenic or tumour-promoting effect of a new IA, comprehensive experimental investigations of the effects of IAs on normal and malignant cells are recommended. In addition to the standard package of pharmacology, toxicology and safety pharmacology studies, recommendations for some special studies are given for innovative IAs. The receptor binding profile of a new IA should be thoroughly characterized. As the minimum it is recommended to study the effects on insulin receptor and IGF-1R. The ligands human insulin, IGF-1, and AspB10 should be included as controls in all experiments. The quantitative comparison of the mitogenic versus the metabolic activity of an innovative IA is important for the overall conclusion on its biological effects. Always in comparison with human insulin, IGF-1, and AspB10, the metabolic activity should be studied in an assay that has been shown to be most sensitive for effects on the carbohydrate metabolism. In parallel, the mitogenic activity should be studied in an assay that has been shown to be most sensitive for effects on cell proliferation. Finally, the effects on carbohydrate metabolism and cell proliferation should be assessed simultaneously in cells that react to both the mitogenic and the metabolic properties of the IA. It is acceptable, that such cells may be less sensitive to either of the effects.

For a new IA the exact mechanism of carcinogenicity (if any) may be unknown. The IGF1-R mediated increase in cell proliferation may not be the only mechanism by which IAs potentially induce or promote cancer. Therefore, studies in cell culture may not be sufficient. Additional studies in tissues (ex vivo) may be integrated in the standard toxicology studies e.g. in the repeated dose toxicity studies. The selection of strain and species is crucial for the relevance of these studies for humans. It is recommended to use a species or strain for which the mitogenic/metabolic potency ratio is similar to man.

It has been recognized that normal and malignant cells or tissues may react differently to IAs. Additional studies with neoplastic tissues may be helpful and should be given consideration on a case by case basis although this approach clearly exceeds the classical approach in toxicological studies using healthy animals.

## The interface between regulatory and health technology assessment

The demonstration of efficacy, safety and pharmaceutical quality may be sufficient to attain the European marketing authorization. This may, however, not be equal to the factual access of patients to the new product as pricing and reimbursement decisions depend on national or regional health systems [20]. It has become increasingly difficult within the European Union to explain divergent assessments and decisions by health technology assessment bodies and regulatory agencies. In the public perception, a positive opinion of the regulators on an innovative new product and the subsequent denial of reimbursement by health technology assessment bodies are contradictory. To minimize differences in the assessments of regulators and health technology assessment bodies, the European network for Health Technology Assessment (EUnetHTA) and the European Medicines Agency started collaboration on European Public Assessment Report contribution to relative effectiveness assessment in February 2010 [21]. By now, the contents and structure of regulators' assessment reports and most particularly the benefit risk section has been modified to better suit the health technology assessment bodies' needs.

The new structure of European Public Assessment Reports (Table 1) is meant to facilitate the use of the assessment by health technology assessment bodies. In the European Public Assessment Reports beneficial and unfavourable effects will be quantitatively described and limitations and uncertainties are given. The report will weight the beneficial and unfavourable effects, give their relative importance and describe possible trade-offs.

**Table 1:** New Structure of European Regulatory Assessment Reports for Benefit Risk Section

step/task	treatment effects	level of uncertainties
review of benefits	favourable effects: beneficial effects, focussed on but not limited to clinical efficacy	uncertainty: impact of supportive data, subgroup differences, assumptions and expectations

review of risks	unfavourable effects: adverse drug reactions, drug-drug interactions, toxicity profile, potential for misuse, environmental impact	uncertainty: trial specifics, e.g. limited no. of patients, above average supervision in trials, non-clinical safety findings

value assignment	compare and rank the favourable effects among each other compare and rank the unfavourable effects among each other	discuss impact of uncertainties on ranking of favourable effects discuss impact of uncertainties on ranking of unfavourable effects

Assessment of benefit-risk balance	trade-off favours versus unfavours and discuss the benefit-risk balance critically	discuss uncertainties in benefit-risk balance discuss values according to perspectives of different stakeholders

The new standardized description will be an integral part of the assessment report for any new application. This will lead to the development of a system of clearly assigned values and trade-offs which over time may develop into a European database for weighted relative benefits. This data base may be used by health technology assessment bodies all over Europe for value-based price finding providing a generally accepted understanding on benefits and risks as a common basis to which the specifics of the national or regional health care system or economics are added. Therefore, applicants may want to take this aspect - price and reimbursement - into consideration when preparing their European marketing authorization application.

To foster the early alignment of different stakeholders, joint scientific advice procedures by regulators and health technology assessment bodies are developed on European or national level [22-24]. This is a promising way to avoid European marketing authorization decisions that have little or no consequence for patients who do not have access to the licensed product for economic reasons.

This sort of exchange, collaboration and ultimately alignment may be a clear win-win situation: patients and their physicians would certainly welcome if clinical practice and international treatment guidelines and recommendations were better in line with regulatory and reimbursement decisions. Health technology assessment bodies and payers could better benefit from the regulators work and more easily build their own assessment on top of the regulators' assessment. To facilitate this, their needs with regard to structure and contents of the regulators assessment reports must be met. Industry should be interested in a better predictability of regulators' and health technology assessment bodies' decisions.

Whereas this alignment process may be relevant for all innovative medicinal products, it may be particularly relevant in the ongoing discussion on the price and reimbursement for IAs in important European markets [25].

## Conclusions

For innovative IAs specific regulatory guidance for European marketing authorization application is available. In addition to the basic demonstration of efficacy and safety, particular consideration is given to a potential cancer hazard. Additional studies exceeding the usual safety package that may address this issue have been suggested by the European Medicines Agency. Combined or joint scientific advice procedures from regulators and health technology assessment bodies are developing on the European level and on a national level in several European Member States.

## Competing interests

The authors declare that they have no competing interests.

## Authors' contributions

HE drafted the manuscript. MW contributed substantially during preparation particularly on the appropriate inclusion of regulatory guidelines. The draft was jointly finalised by HE and MW and all authors read and approved the final manuscript.
